# Lipolysis-Stimulated Lipoprotein Receptor Impairs Hepatocellular Carcinoma and Inhibits the Oncogenic Activity of YAP1 *via* PPPY Motif

**DOI:** 10.3389/fonc.2022.896412

**Published:** 2022-05-02

**Authors:** Xin Dong, Xianbin Zhang, Peng Liu, Yu Tian, Li Li, Peng Gong

**Affiliations:** ^1^ Department of Hepatobiliary Surgery, The First Affiliated Hospital of Dalian Medical University, Dalian, China; ^2^ Department of General Surgery & Institute of Precision Diagnosis and Treatment of Gastrointestinal Tumors, Shenzhen University General Hospital & Shenzhen University Clinical Medical Academy, Shenzhen, China; ^3^ Carson International Cancer Center & Guangdong Provincial Key Laboratory of Regional Immunity and Diseases, Shenzhen University Health Science Center, Shenzhen, China; ^4^ Guangdong Provincial Key Laboratory for Biomedical Measurements and Ultrasound Imaging, School of Biomedical Engineering, Shenzhen University Health Science Center, Shenzhen, China

**Keywords:** lipolysis-stimulated lipoprotein receptor, hepatocellular carcinoma, YAP1, PPPY motif, Hippo

## Abstract

**Purpose:**

Lipolysis-stimulated lipoprotein receptor (LSR) is a type I single-pass transmembrane protein which is mainly expressed in the liver. In this study, we investigated if and how LSR is involved in the carcinogenesis of hepatocellular carcinoma (HCC).

**Experimental Design:**

To evaluate if LSR was abnormally expressed in human HCC tissues, and how its expression was associated with the survival probability of patients, we obtained data from Gene Expression Omnibus and The Cancer Genome Atlas Program. To investigate if and how LSR regulates tumor growth, we knocked down and overexpressed LSR in human HCC cell lines. In addition, to evaluate the interaction between LSR and yes-associated protein1 (YAP1), we mutated LSR at PPPY motif, a binding site of YAP1.

**Results:**

Totally, 454 patients were enrolled in the present study, and high expression of LSR significantly decreased the probability of death. Knockdown of LSR significantly increased the expansion of HCC cells and significantly promoted tumor growth. In addition, downregulation of LSR increased the nuclear accumulation and transcriptional function of YAP1. Conversely, overexpression of LSR impairs this function of YAP1 and phosphorylates YAP1 at serine 127. Of note, mutation of LSR at the PPPY motif could block the interaction between LSR and YAP1, and restore the transcriptional ability of YAP1.

**Conclusions:**

The present study suggests that LSR binds to YAP1 *via* the PPPY motif. Thus, LSR increases the phosphorylation of YAP1 and impairs the growth of HCC. This highlights that targeting LSR might be a promising therapeutic strategy for HCC.

## Introduction

Hepatocellular carcinoma (HCC) accounts for between 85% and 90% of primary liver cancers (1–3). It seriously endangers public health in Japan (4), China (5), United States (6), and several European regions (7). Unfortunately, systemic chemotherapy failed to improve the survival of advanced HCC patients (8). Sorafenib, a molecular targeting Raf kinases, could give rise to survival benefits for patients with advanced HCC (9). But it only slightly improved the median overall survival (sorafenib vs. placebo: 10.7 months vs. 7.9 months) (9). Thus, there is an urgent need to identify new therapeutic targets and develop novel treatment strategies for HCC.

Lipolysis-stimulated lipoprotein receptor (LSR) is a type I single-pass transmembrane protein, which is mainly expressed in the liver (10, 11). Interestingly, some studies recently suggested that LSR is involved in tumor initiation and progression (12–15). Moreover, other studies reported that LSR impairs different aspects of cancer pathophysiology such as invasive growth (16, 17) and enhances chemosensitivity (18). Unfortunately, the effect of LSR in HCC is still unclear.

Yes-associated protein1 (YAP1) is a core component of the Hippo signaling pathway, which is involved in oncogenesis (19) and might be a promising target for treating HCC (20, 21). It has been demonstrated that YAP1 can move to the nucleus and promote the transcription of several genes, such as *cysteine-rich 61* (*CYR61*) and connective tissue growth factor (*CTGF*), which promote tumor growth (22, 23). However large tumor suppressor kinases (LATS1 and LATS2, LATS1/2) can phosphorylate YAP1 at serine 127 and retain YAP1 in the cytoplasm (19). In this case, YAP1 cannot translocate to the nucleus to induce the transcription of its target genes (24). Interestingly, it has been reported that knockdown of LSR could upregulate the expression of *CYR61*, the target gene of YAP1 (25). This suggests that LSR might be also involved in regulating the function of YAP1.

Thus, the present study evaluated the expression of LSR in human HCC tissues and investigated the effects of LSR *in vitro* and *in vivo*. In addition, we also investigated if and how LSR regulated the function of YAP1.

## Materials and Methods

### Human Tissues, Cell Culture and Antibodies

**Figure 1:**
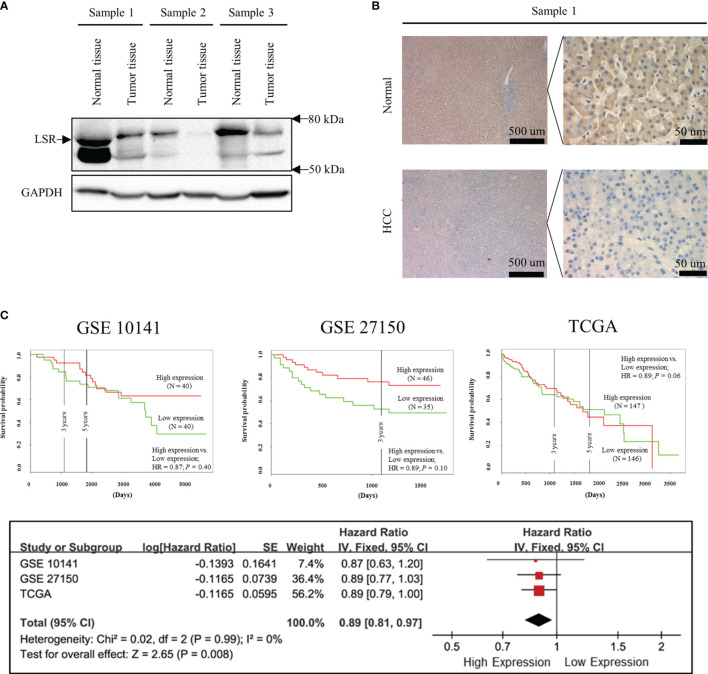
Low expression of LSR increases the risk of death. Immunoblotting **(A)** and immunohistochemistry **(B)** experiments suggest that LSR is highly expressed in liver but not in HCC tissues. Kaplan-Meier curves **(C)** were obtained from data deposited in the Gene Expression Omnibus (GEO, accession number: GSE 10143 and GSE 27150) and The Cancer Genome Atlas (TCGA) data bank with the help of PROGgeneV2 (http://genomics.jefferson.edu/proggene/). The synthesis of hazard ratios were performed using Review Manager and demonstrated that high expression of LSR significantly reduced the risk of death.

The human tissues, in **Figures 1A, B**, were obtained from patients who underwent hepatectomy in the First Affiliated Hospital of Dalian Medical University. The protocol of this study was approved by the Ethics Committee of the First Affiliated Hospital of Dalian Medical University in accordance with the declaration of Helsinki. The human HCC cell lines, Hep3B, Huh7, SNU449, and embryonic kidney 293T cells were purchased from ATCC (Manassas, USA). The SNU449 cells were cultured in RPMI 1640 medium. The Huh7 and 293T cells were cultured in Dulbecco’s modified eagle medium (DMEM) and the Hep3B cells were cultured in Eagle’s minimal essential medium (EMEM). The media were supplemented with 10% fetal bovine serum, 100 U/ml penicillin and 100 μg/ml streptomycin. The cells grew in a humidified 5% CO_2_ incubator at 37°C, and the proteins were measured with the antibodies as specified in **Supplementary Table 1**.

### Clonogenic Assay and Xenograft Model

**Figure 2:**
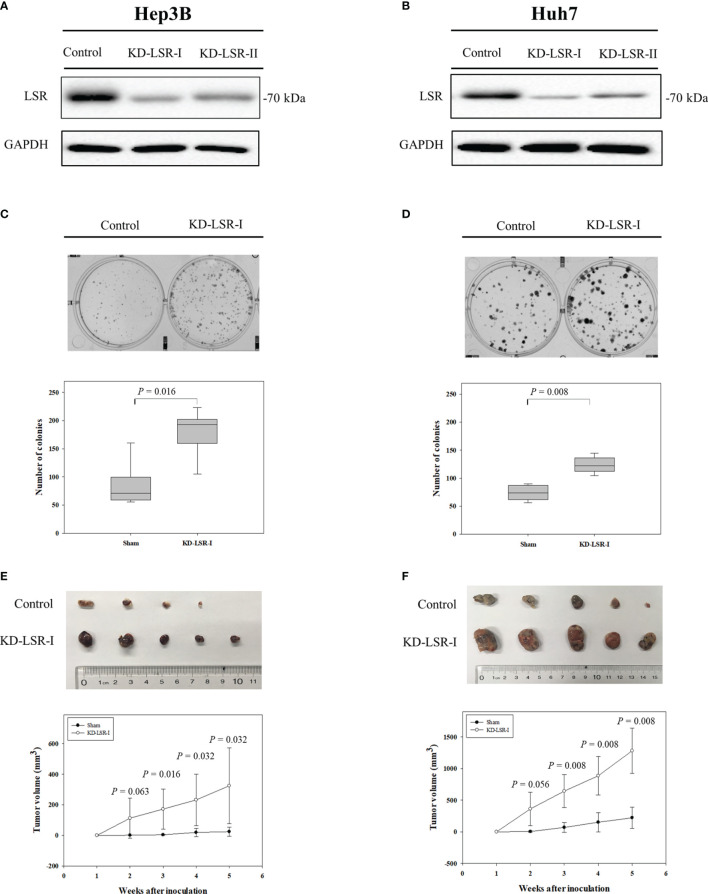
Knockdown of LSR promotes the expansion of HCC cells and increases tumor growth. Knockdown of LSR in Hep3B **(A)** and Huh7 **(B)** cells significantly increased the expansion of these cells **(C, D)**, and significantly enhanced tumor growth *in vivo* **(E, F)**. The significances of differences were evaluated by Mann-Whitney U test. For C and D, the experiments were independently repeated six times. For **(E, F)** the mean ± standard deviation was obtained from nine mice and ten mice.

For the clonogenic assay in **Figure 2**, 1 ×10^3^ Hep3B cells or Huh7 cells per well were seeded into 6-well plates. After 14 days, the cells were stained with 0.2% crystal violet (Damao, Tianjin, China) and the number of colonies, consisting of 50 or more cells, were determined with the help of a Leica DMI4000B microscopy (Leica, Mannheim, Germany). To evaluate the effect of LSR *in vivo*, six-week-old female BALB/c (CAnN.Cg-Foxn1^nu^/Crl) nude mice were purchased from Charles River (Wilmington, MA, USA) and bred in the central animal facility of the Dalian Medical University as previously described (26). 2 × 10^6^ HCC cells or the identical number of LSR knockdown cells were resuspended in 100 µL phosphate-buffered saline and subcutaneously injected into the right flank of the BALB/c nude mice. Tumor volume was determined by digital calipers weekly and calculated using the formula: π/6 × large diameter × small diameter2 (27). The *in vivo* study was approved by the Animal Experimentation Ethics Committee of Dalian Medical University (Approval No. AEE17027).

### Immunofluorescence and Immunohistochemistry Assay

**Figure 3:**
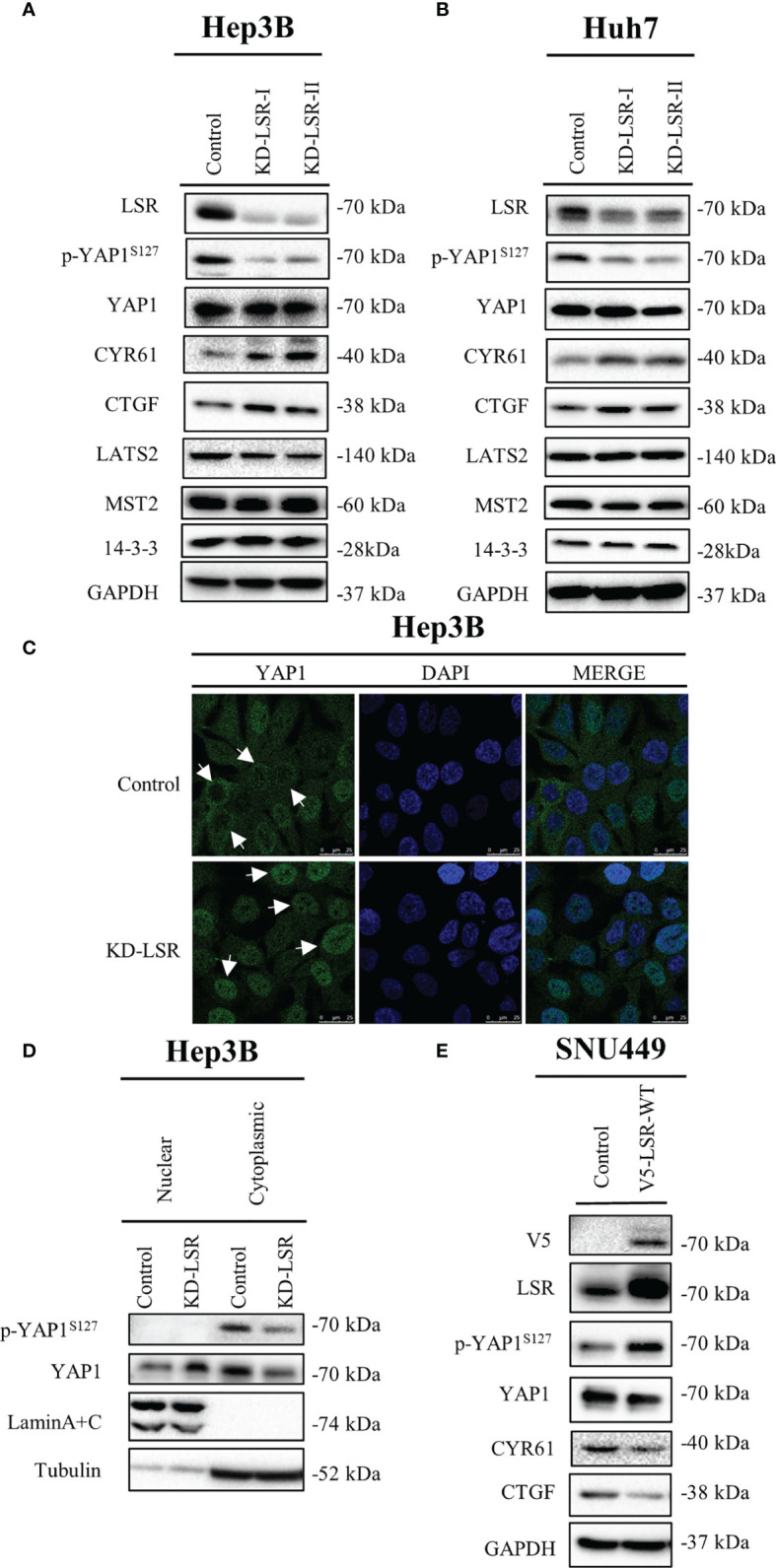
Knockdown of LSR reduces phosphorylation of YAP1 at serine 127 and promotes nuclear localization of YAP1. Knockdown of LSR decreased the accumulation of phosphorylated YAP1 and increased the level of CYR61 and CTGF in Hep3B **(A)** and Huh7 **(B)** cells. In addition, downregulation of LSR promoted YAP1 to move into nucleus **(C, D)**. Conversely, overexpression of LSR increased the level of phosphorylated YAP1 and decreased the accumulation of CYR61 and CTGF in SNU449 cells **(E)**. Bar = 25 µm.

For immunofluorescence in **Figure 3**, 2 × 10^5^ Hep3B cells were seeded in a glass-bottom dish (NEST, Wuxi, China, code 801001). After 24 hours, these cells were incubated with a rabbit-anti-LSR antibody followed by a fluorescence-labeled secondary antibody (**Supplementary Table 1**), and the nuclei were stained by 4’, 6-diamidino-2-phenylindole (DAPI, Sigma-Aldrich, St. Louis, USA, code: D9542). The images were acquired by a confocal microscope, Leica TCS SP5 (Leica, Mannheim, Germany), using the 60× oil objective. For the immunohistochemistry assay, the staining of LSR was performed on 4 μm paraffin sections using a rabbit anti-LSR antibody (**Supplementary Table 1**). In addition, the nuclei were stained by hematoxylin (ZSGB-BIO, Beijing, China).

### Western Blot and Immunoprecipitation

To evaluate the accumulation of proteins in human tissues, the western blots were performed as previously described (28) using the antibodies in **Supplementary Table 1**. In addition, the cell fractionation assay was performed using the NE-PER Kit (Pierce, Rockford, USA, code: 78833). To evaluate if LSR binds to YAP1, immunoprecipitation was performed in 293T cells. Briefly, 1.5 × 10^6^ 293T cells per well were plated in a tissue-culture dish and transfected with distinct plasmids. After 24 hours, the cell lysates were incubated with relevant antibodies for one hour. Subsequently, proteins bound to the antibodies were purified with protein G agarose (Roche; Basle, Switzerland; code: 11243233001) for three hours and western blots were performed.

### Plasmids

**Figure 4:**
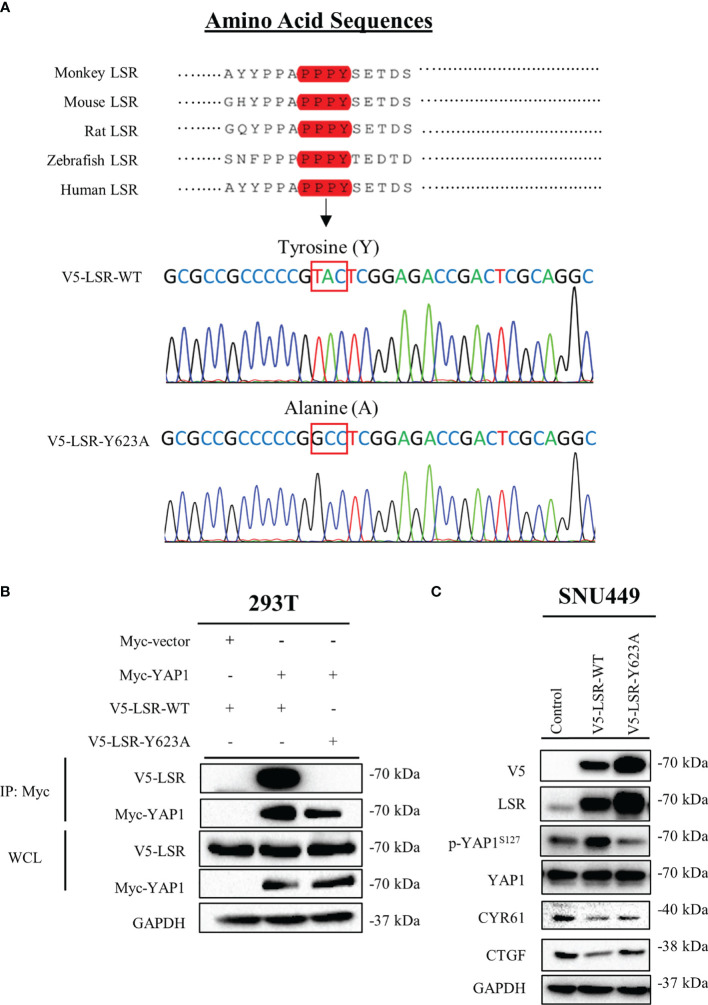
LSR binds to YAP1 *via* a PPPY motif. LSR contains a conserved PPPY motif **(A)**. An antibody directed against the Myc tag of YAP1 could pull down wild type LSR but not a LSR protein, which had a tyrosine to alanine mutation in the PPPY domain **(B)**. Wild type but not mutated LSR induced the phosphorylation of YAP1 and reduced the level of CYR61 and CTGF **(C)**.

The human DNA, pCMV-HA-LSR, was a kind gift from Dr. James Hastie at the University of Dundee, Scotland, United Kingdom (29). To obtain the V5 tagged LSR plasmid (pcDNA3.1-V5-LSR-WT, in **Figures 4A, B**), the pCMV-HA-LSR was amplified by Pfu DNA polymerase (Thermo Fisher Scientific; Waltham, USA; code: EP0502) using the primer specified in **Supplementary Table 2**. Subsequently, the polymerase chain reaction (PCR) fragment of wild-type LSR was inserted between BamH1 and XbaI sites of a pcDNA 3.1/V5-His TOPO vector (Thermo Fisher Scientific; code: K480040). To generate the mutation of V5 tagged LSR (pcDNA3.1-V5-LSR-Y623A, **Figures 4A, B**), a PCR-mediated site-directed mutagenesis was performed using the primers specified in **Supplementary Table 2**. The PCR primer sequences and the DNA sequences were synthesized and verified by GenScript (Nanjing, China). In addition, the plasmid expressing the Myc tagged YAP1 protein (Myc-YAP1, **Figure 4B**) was a gift from Prof. Zengqiang Yuan at the Institute of Biophysics, Beijing, China (30).

### Lentivirus Transfected Cell Lines

To downregulate the expression of LSR in Hep3B and Huh7 cells, these cells were infected by shRNA lentiviral particles (Santa Cruz; code: sc-97082V) in the presence of polybrene and puromycin. Subsequently, two LSR knocked down clones, KD-LSR-I and KD-LSR-II, were used for this study. To obtain the V5-LSR-WT and V5-LSR-Y623A stably overexpressing SNU449 cells, the PCR fragments were inserted between EcoRI and BamHI sites of a pCDH-CMV vector (System Biosciences, California, USA, code: CD510B-1) with the help of the primer defined in in **Supplementary Table 2**. Subsequently, 293T cells were transfected by pCDH-V5-LSR-WT or pCDH-V5-LSR-Y623A in the presence of the lentiviral packaging plasmid, psPAX2 and pMD2.G, using Lipofectamine 3000 (Thermo Fisher Scientific; code: L3000001). psPAX2 was a gift from Didier Trono (Addgene; Watertown, Massachusetts, USA; code: 12260) and pMD2.G was a gift from Didier Trono (Addgene; code: 12259). After 48 hours, the supernatants were collected and filtered with a filter (0.45 µm pore size), and used for infecting the SNU449 cells in the presence of 8 μg/mL polybrene (Santa cruz, Texas; USA; code: sc-134220) for 48 hours. Then these SNU449 cells were cultured in the medium containing 5 ug/mL puromycin (Amresco, Pennsylvania, USA, code: J593) for the selection of stable clones.

### Statistical Analysis

In order to calculate the hazard ratio (HR) in **Figure 1C**, the data were obtained from Gene Expression Omnibus (GEO, accession number: GSE 10143 and GSE 27150) and The Cancer Genome Atlas (TCGA) Program with the help of PROGgeneV2 (http://genomics.jefferson.edu/proggene/) (31). The synthesis of data was performed as previously described (32) using Review Manager (Computer program. Version 5.3. Copenhagen: The Nordic Cochrane Centre, The Cochrane Collaboration, 2014). For the clonogenic assay (**Figures 2C, D**), the data were presented as box plots, and for tumor volume (**Figures 2E, F**), the data were expressed as mean ± standard deviation. The significance of differences were evaluated by the Mann-Whitney U test using SigmaPlot12.0 (SYSTAT Software Inc., San Jose, USA). A *P-*value < 0.05 was considered as a statistically significant.

## Results

### LSR Impairs the HCC

In order to investigate the expression of LSR during carcinogenesis of HCC, we measured the accumulation of LSR in HCC and normal liver tissues by western blot. We observed that the level of LSR was more abundant in the liver than in tumor tissues (**Figure 1A**). This observation was confirmed by immunohistochemistry (**Figure 1B**). In addition, we evaluated, if the abnormal expression of LSR in HCC tissues was associated with an altered survival of patients. We obtained data from three HCC cohorts: GSE 10143, GSE 27150, and TCGA (**Figure 1C**). Importantly, the forest plot of these data suggested that, compared to low expression of LSR, high expression of LSR reduced the risk of death by 11% (high expression of LSR vs. low expression of LSR: HR = 0.89, 95% CI, 0.81 and 0.97, *P =* 0.008, **Figure 1C**). This suggests that LSR might inhibit the development of HCC. To evaluate this hypothesis, we inhibited the expression of LSR in Hep3B (**Figure 2A**) and Huh7 (**Figure 2B**) cells by shRNA. We observed that knockdown of LSR significantly promoted the expansion of these cells (**Figures 2C, D**). In addition, we evaluated control and knocked down cells in a xenograft model and we found that inhibiting the expression of LSR increased the tumor volume (**Figures 2E, F**). These data suggest that LSR impairs the growth of HCC.

### LSR Impairs the Nuclear Localization and Transcriptional Function of YAP1

To evaluate if LSR regulates YAP1 signaling, LSR was depleted in Hep3B (**Figure 3A**) and Huh7 (**Figure 3B**) cells. We observed that the knockdown of LSR impaired the phosphorylation of YAP1 at serine 127, however, it failed to have an influence on the level of YAP1 and LATS2, a major kinase that phosphorylates YAP1 at serine 127 (**Figures 3A, B**). In addition, downregulation of LSR increased the accumulation of CYR61 and CTGF, two target proteins of YAP1 (**Figures 3A, B**). This suggests that knockdown of LSR might increase the nuclear localization of YAP1 and promote the transcription of *CYR61* and *CTGF*. To evaluate this hypothesis, we investigated the localization of YAP1 by immunofluorescence assay and cell fractionation experiments. Indeed, knockdown of LSR promoted the nuclear localization of YAP1 and reduced the cytoplasmic YAP1 concentration (**Figures 3C, D**). Moreover, we also investigated if overexpression of LSR could promote the phosphorylation of YAP1 and impair the transcriptional function of YAP1. We transduced LSR wild-type (V5-LSR-WT) into SNU449 cells. We observed that overexpression of LSR could increase the accumulation of phosphorylated YAP1 and decreased the level of CYR61 and CTGF (**Figure 3D**). This confirms that LSR might inhibit the nuclear accumulation of YAP1. Taken together, knockdown and overexpression of LSR imply that this protein enhances the phosphorylation of YAP1 at serine 127 and impairs the nuclear localization and function of YAP1.

### LSR Binds to YAP1 *via* a PPPY Motif and Inhibits the Transcriptional Ability of YAP1

**Figure 5:**
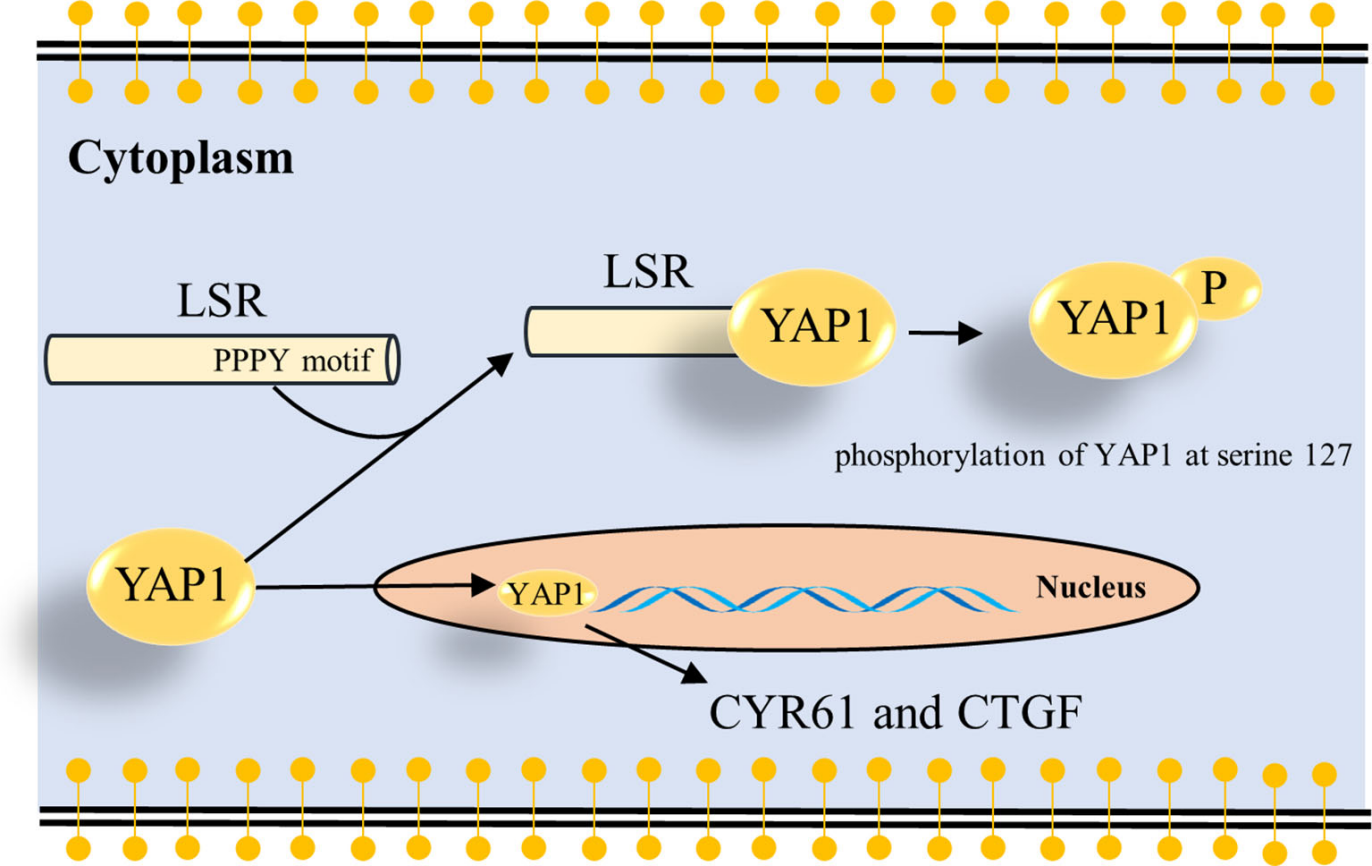
Schema how LSR regulates YAP signaling. The present study suggests that LSR binds to YAP1 *via* the PPPY motif and induces the phosphorylation of YAP1 at serine 127 (**Figure 4**). This leads to the cytoplasmic retention of YAP1 and impairs the oncogenic function of YAP1 in the nucleus. As a result the transcription of YAP target genes, *CYR61* and *CTGF*, is reduced.

To understand how LSR impairs the function of YAP1, we investigated different domains of LSR. We observed that LSR contains a conserved PPxY motif, PPPY (**Figure 4A**). It has been demonstrated that a peptide containing the PPxY motif could bind to the WW domain of YAP1 (33–35). Thus we assumed that LSR could bind to YAP1 directly. In order to verify this hypothesis, the Myc tagged YAP1 (Myc-YAP1) and V5 tagged LSR (V5-LSR-WT) were transfected into 293T cells and the interaction between these two proteins was measured by a pull-down assay. Indeed, LSR could be pulled down by an antibody directed against the Myc tag of YAP1 (**Figure 4B**). In order to evaluate if the PPPY motif of LSR is responsible for the interaction between LSR and YAP1, we transfected the mutated LSR (V5- LSR-Y623A, **Figure 4A**) and Myc-YAP1 into 293T cells. We observed that the interaction between LSR and YAP1 was abolished by the Y623A mutation (**Figure 4B**). This suggests that LSR binds to YAP1 *via* the PPPY motif. In addition, wild-type LSR, V5-LSR-WT, increased, whereas the mutated LSR, V5-LSR-Y623A, decreased the accumulation of phosphorylated YAP1 (**Figure 4C**). Moreover, only wild-type LSR but not the mutated LSR reduced the accumulation of CYR61 and CTGF (**Figure 4C**). Based on these data, we speculate that LSR indeed interacts with YAP1 *via* the PPPY motif and impairs the transcriptional function of YAP1 (**Figure 5**).

## Discussion

In the present study, we observed that LSR induces the phosphorylation of the oncogene YAP1 at serine 127 and blocks the nuclear translocation and transcriptional function of YAP1 (**Figures 3**, **4**). Since LSR is not a kinase, and thus cannot directly phosphorylate YAP1, this observation could be explained by the assumption that LSR also binds directly or indirectly to LATS1/2, two kinases phosphorylating YAP1 at serine127 (19). Subsequently, LSR might help to associate LATS1/2 to YAP1, which then facilitates the phosphorylation of YAP1 by LATS1/2 (36). Thus LSR could be a scaffolding protein with a similar function as other scaffolding proteins, for example axin (37), which has been demonstrated to suppress the growth of HCC cells (38). Previous studies suggest that LSR contributes to clear the atherogenic triglyceride-rich lipoproteins and low-density lipoproteins (10, 13, 14). This implies that the lipid metabolic might be involved in the interaction between LSR and YAP1; and additional study need to address this question. Consistent with our study, Shimada et al. proved that LSR impairs cancer cells (17, 39). In addition, Shimada et al. observed that the level of LSR in endometrial cancer tissues was low and YAP1 was accumulated in the nucleus (39). This suggests that down regulation of LSR might promote the nuclear localization of YAP1. However, Shimada et al. reported that LSR increased the level of phosphorylated YAP1, which reduced the nuclear localization of YAP1 (39). Therefore, how LSR regulates YAP1, whether to promote the accumulation of YAP1 in the nucleus or the retention of YAP1 in the cytoplasm, still needs to be further studied.

An anti-oncogenic function of LSR was also observed in a study, which demonstrated that abolished LSR could increase the motility and invasion of bladder cancer cells (25). In addition, this study demonstrated that knockdown of LSR could increase the expression of pro-oncogenic genes, such as *interleukin 1 alpha* and *endothelin 1*, which could contribute to tumor initiation and progression (40, 41).

In contrast to these publications, other studies demonstrated that LSR was overexpressed in breast and gastric cancers (42, 43) and that overexpressed LSR in breast cancer cells causes the development of cancer in xenograft studies (44). In addition, the knockdown of LSR could also increase the expression of the anti-oncogenic gene, *early growth response 1*, which could upregulate the accumulation of Bcl-2-associated X protein (BAX), a pro-apoptotic protein (45).

These contradictory studies suggest that LSR might have pro- and anti-cancerous effects (17, 25, 39, 42–44). This raises the following issue: Should the function of LSR be inhibited or induced when treating cancer patients? To answer this question it will be important to investigate the expression of LSR in individual cancer types and to evaluate if expression levels are associated with poor or good survival. This information, in addition to gain and loss of functions experiments, is crucial for deciding if one should activate or inactivate LSR function in cancer patients.

The present study demonstrated that high expression of LSR decreased the risk of death for HCC patients and also *in vitro* and *in vivo* studies demonstrate an anti-oncogenic function of LSR. This suggests that overexpression of the *LSR* gene might be a promising strategy to treat HCC. An optimal transportation of this gene therapy might be adenovirus, which has been approved by the Chinese State Food and Drug Administration for treating head and neck cancer (46). In addition, a clinical study has proved that treating patients with the adenoviral vector-mediated *IFN-Alpha 2b* is safe in malignant mesothelioma patients, and this gene therapy in combination with traditional chemotherapies increased the overall survival rate, when compared to patients treated only by chemotherapies (47). Thus, a preclinical study, which will evaluate the anticancer efficacy of adenoviral vector-mediated *LSR* in combination with sorafenib, the first line targeted therapeutic drug of HCC, might allow us to understand the benefit of treating HCC by targeting LSR.

## Conclusions

In conclusion, the present study proposes that high expression of LSR decreases the probability of death in human HCC cohorts. *In vitro* and *in vivo* experiments also demonstrate that LSR inhibits HCC cell expansion and reduced tumor growth. We also observed that LSR binds to YAP1 and impairs the oncogenic function of YAP1. This suggests that LSR might be a promising target for developing a novel therapy for HCC patients.

## Data Availability Statement

The datasets presented in this study can be found in online repositories. The names of the repository/repositories and accession number(s) can be found in the article/**Supplementary Material**.

## Ethics Statement

The studies involving human participants were reviewed and approved by Ethics Committee of the First Affiliated Hospital of Dalian Medical University. The patients/participants provided their written informed consent to participate in this study. The animal study was reviewed and approved by Ethics Committee of Dalian Medical University. Written informed consent was obtained from the individual(s) for the publication of any potentially identifiable images or data included in this article.

## Author Contributions

Conception and design: PG, XZ, and LL. Development of methodology: XD. Acquisition of data (provided animals, acquired and managed patients, provided facilities, etc.): XD and XZ (**Figure 1C**). Analysis and interpretation of data (e.g., statistical analysis, biostatistics, computational analysis): XD, XZ, and PL. XZ wrote the first version of this manuscript and all authors revised and approved the last version of this manuscript.

## Funding

The study was supported by the Natural Science Foundation of Shenzhen University General Hospital (Grant number: SUGH2018QD050); Shenzhen Science and Technology Innovation Commission (Grant number: RCBS20200714114958333 and JCYJ20190808114203755); Shenzhen Key Medical Discipline Construction Fund & Sanming Project of Medicine in Shenzhen (SZSM202111002); The Project of Department of Education of Guangdong Province (2020KZDZX1170); Guangdong Basic and Applied Basic Research Fund (2020A1515110083).

## Conflict of Interest

The authors declare that the research was conducted in the absence of any commercial or financial relationships that could be construed as a potential conflict of interest.

## Publisher’s Note

All claims expressed in this article are solely those of the authors and do not necessarily represent those of their affiliated organizations, or those of the publisher, the editors and the reviewers. Any product that may be evaluated in this article, or claim that may be made by its manufacturer, is not guaranteed or endorsed by the publisher.
