# Supplementary material for: Lipolysis-Stimulated Lipoprotein Receptor Impairs Hepatocellular Carcinoma and Inhibits the Oncogenic Activity of YAP1 via PPPY Motif
Source: Front Oncol. 2022 May 2;12:896412. doi: 10.3389/fonc.2022.896412 (PMC9108500; doi:10.3389/fonc.2022.896412)
Supplement: Supplementary file 1 [file Table_1.docx]

**Supplementary Table 1. The antibodies used in the present study**

| **Primary antibodies** | **Dilution** |
| --- | --- |
| Rabbit anti-LSR  (Cell Signaling, Danvers, USA, code: 14804S) | WB (1000×)  IHC (400×) |
| Rabbit anti-LATS2  (Cell Signaling, Danvers, USA, code: 5888S) | WB (1000×) |
| Rabbit anti-YAP1  (Novus, [Centennial, Colorado, USA](https://www.google.com/search?client=firefox-b-ab&q=Centennial,+Colorado&stick=H4sIAAAAAAAAAOPgE-LSz9U3sEw3N48vU-IEsY3KKjJMtTQyyq30k_NzclKTSzLz8_Tzi9IT8zKrEkGcYqv0xKKizGKgcEbhIlYR59S8ktS8vMzEHB0F5_yc_KLElHwAJqZDSloAAAA&sa=X&ved=2ahUKEwiR4_O7yP_hAhUN2aQKHUyLDuIQmxMoATAQegQIDBAH), code: NB110-58358) | WB (1000×)  IF (400×) |
| Rabbit anti-p-YAP1  (Cell Signaling, Danvers, USA, code:4911S) | WB (1000×) |
| Mouse anti-CYR61  (Santa cruz, [Texas, USA](https://www.google.com/search?client=firefox-b-ab&sa=X&biw=1440&bih=764&q=Dallas&stick=H4sIAAAAAAAAAOPgE-LUz9U3SDPOTUpT4gAzjYoKtbSyk63084vSE_MyqxJLMvPzUDhWGamJKYWliUUlqUXFi1jZXBJzchKLAbkSuxtMAAAA&ved=2ahUKEwiO-puQyv_hAhXH16QKHSwWA7gQmxMoATATegQICxAH), code: sc-13100) | WB (200×) |
| Rabbit anti-CTGF  (Abcam, [Cambridge, United Kingdom](https://www.google.com/search?client=firefox-b-ab&biw=1440&bih=764&q=Cambridge&stick=H4sIAAAAAAAAAOPgE-LSz9U3ME0ySC43VOIAsS3NLYq0tLKTrfTzi9IT8zKrEksy8_NQOFYZqYkphaWJRSWpRcWLWDmdE3OTijJT0lMBPI_o5lAAAAA&sa=X&ved=2ahUKEwjglrDyyv_hAhUB2KQKHd1hCAYQmxMoATAVegQIChAL), code: ab6992) | WB (1000×) |
| Rabbit anti-Lamin A+C  (Abcam, [Cambridge, United Kingdom](https://www.google.com/search?client=firefox-b-ab&biw=1440&bih=764&q=Cambridge&stick=H4sIAAAAAAAAAOPgE-LSz9U3ME0ySC43VOIAsS3NLYq0tLKTrfTzi9IT8zKrEksy8_NQOFYZqYkphaWJRSWpRcWLWDmdE3OTijJT0lMBPI_o5lAAAAA&sa=X&ved=2ahUKEwjglrDyyv_hAhUB2KQKHd1hCAYQmxMoATAVegQIChAL), code: ab133256) | WB (10000×) |
| Mouse anti-Tubulin (Sigma, [[St](file:///D:\program%20use\Dict\7.0.1.0214\resultui\dict\result.html?keyword=St) [Louis](file:///D:\program%20use\Dict\7.0.1.0214\resultui\dict\result.html?keyword=Louis), USA](https://www.google.com/search?client=firefox-b-ab&biw=1440&bih=764&q=Kawasaki,+Kanagawa&stick=H4sIAAAAAAAAAOPgE-LUz9U3MMlIMjZSAjPTC8zM8rS0spOt9POL0hPzMqsSSzLz81A4VhmpiSmFpYlFJalFxYtYhbwTyxOLE7MzdRS8E_MS04E8AI36IalZAAAA&sa=X&ved=2ahUKEwjIzquWzP_hAhXCyKQKHQuVDCgQmxMoATAZegQIDhAL) , code: 85102247) | WB (20000×) |
| Rabbit anti- GAPDH  (Proteintech, [Chicago](file:///D:\program%20use\Dict\7.0.1.0214\resultui\dict\result.html?keyword=Chicago) , USA, code: 10494-1-AP) | WB (80000×) |
| Mouse anti-Myc  (Invitrogen,California, USA,code:132500) | IP (50×) |
| Mouse anti-V5  (Proteintech, [Chicago](file:///D:\program%20use\Dict\7.0.1.0214\resultui\dict\result.html?keyword=Chicago) , USA,code: 66007-I-Ig) | WB (5000×) |
| **Secondary antibodies** |  |
| Goat anti-rabbit HRP conjugated  (Santa cruz, [Texas, USA](https://www.google.com/search?client=firefox-b-ab&sa=X&biw=1440&bih=764&q=Dallas&stick=H4sIAAAAAAAAAOPgE-LUz9U3SDPOTUpT4gAzjYoKtbSyk63084vSE_MyqxJLMvPzUDhWGamJKYWliUUlqUXFi1jZXBJzchKLAbkSuxtMAAAA&ved=2ahUKEwiO-puQyv_hAhXH16QKHSwWA7gQmxMoATATegQICxAH), code: sc-2030) | WB (40000×) |
| Goat anti-mouse HRP conjugated  (Santa cruz, [Texas, USA](https://www.google.com/search?client=firefox-b-ab&sa=X&biw=1440&bih=764&q=Dallas&stick=H4sIAAAAAAAAAOPgE-LUz9U3SDPOTUpT4gAzjYoKtbSyk63084vSE_MyqxJLMvPzUDhWGamJKYWliUUlqUXFi1jZXBJzchKLAbkSuxtMAAAA&ved=2ahUKEwiO-puQyv_hAhXH16QKHSwWA7gQmxMoATATegQICxAH), code: sc-2005) | WB (40000×) |
| Alexa 488 (green)-conjugated Goat anti-rabbit  (Thermo Fisher Scientific, [Massachusetts, USA](https://www.google.com/search?client=firefox-b-ab&biw=1440&bih=764&q=Waltham,+Massachusetts&stick=H4sIAAAAAAAAAOPgE-LSz9U3MCooMTBJU-IAsTOqjE21tLKTrfTzi9IT8zKrEksy8_NQOFYZqYkphaWJRSWpRcWLWMXCE3NKMhJzdRR8E4uLE5MzSotTS0qKAbi_f6RdAAAA&sa=X&ved=2ahUKEwjnuZij0__hAhWNblAKHTCiBQUQmxMoATAdegQIDhAH), code: A27034) | IF (500×) |

WB, western blot; IHC, immunohistochemistry; IP, immunoprecipitation, IF, immunofluorescence; HRP, horseradish peroxidase.

**Supplementary Table 2. The oligonucleotides of primers in this study**

| Primer | | Oligonucleotides |
| --- | --- | --- |
| pcDNA3.1-V5-LSR | Forward | 5’-CGGGATCCATGCAACAGGACGGACTTGGAGTAG-3’ |
|  | Reverse | 5’-GCTCTAGACTGACGACTAAACTTTCCCGAC-3’ |
| V5-LSR-Y623A | Forward | 5’-CCCGCCCGCGCCGCCCCCGGCCTCGGAGACCG-3’ |
|  | Reverse | 5’-GCCTGCGAGTCGGTCTCCGAGGCCGGGGGCGGC-3’ |
| pCDH-V5-LSR | Forward | 5’-CGGAATTCGCCACCATGCAACAGGACGGACTTGGAGTAG-3’ |
|  | Reverse | 5’-CGGGATCCCTACGTAGAATCGAGACCGAGGAGAGG-3’ |
